# Di-μ_1,1_-azido-bis­[azido­(5,5′-dimethyl-2,2′-bipyridine)nickel(II)]

**DOI:** 10.1107/S1600536808037902

**Published:** 2008-11-20

**Authors:** Jin Hou

**Affiliations:** a Department of Chemistry and Chemical Engineering, South-East University, Nanjing 211189, and Nantong Entry–Exit Inspection and Quarantine Bureau, Nantong 226005, People’s Republic of China

## Abstract

In the title azide-bridged dinuclear centrosymmetric nickel(II) complex, [Ni_2_(N_3_)_4_(C_12_H_12_N_2_)_2_], the Ni^II^ atom is five-coordinated by two N atoms of the 5,5′-dimethyl-2,2′-bipyridine ligand and three N atoms from three azide ligands in a distorted trigonal–bipyramidal geometry. The Ni⋯Ni distance is 3.2398 (12) Å.

## Related literature

For general background, see: Abramo *et al.* (2002 ▶); Dey *et al.* (2007 ▶); Jiang *et al.* (2005 ▶). For related structures, see: Fu *et al.* (2005 ▶); Song *et al.* (2007 ▶).
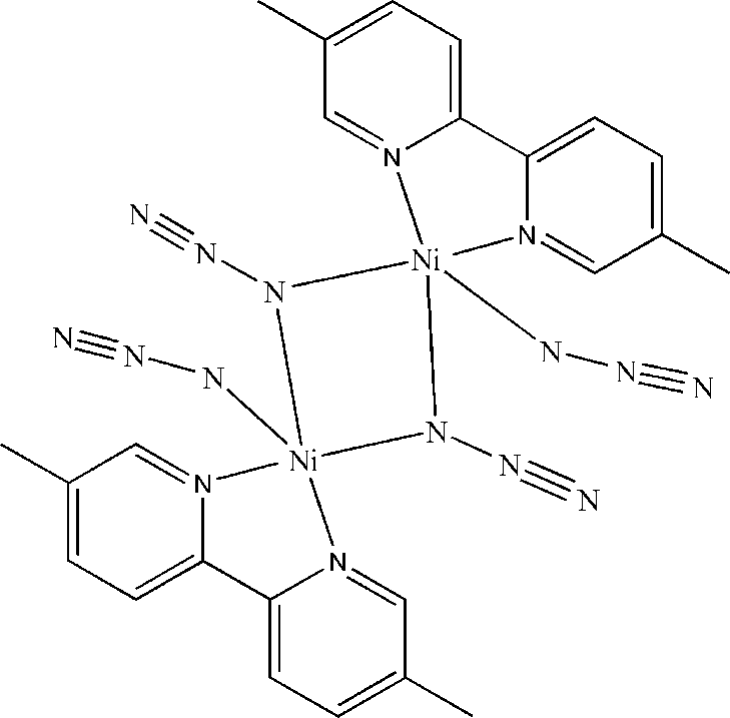


## Experimental

### 

#### Crystal data

[Ni_2_(N_3_)_4_(C_12_H_12_N_2_)_2_]
*M* *_r_* = 654.01
Monoclinic, 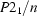

*a* = 7.938 (2) Å
*b* = 15.067 (3) Å
*c* = 11.755 (2) Å
β = 91.650 (2)°
*V* = 1405.3 (5) Å^3^
*Z* = 2
Mo *K*α radiation
μ = 1.39 mm^−1^
*T* = 298 (2) K
0.13 × 0.10 × 0.08 mm

#### Data collection

Bruker SMART CCD area-detector diffractometer
Absorption correction: multi-scan (*SADABS*; Bruker, 2001 ▶) *T*_min_ = 0.840, *T*_max_ = 0.897
11588 measured reflections
3060 independent reflections
2165 reflections with *I* > 2σ(*I*)
*R*_int_ = 0.063

#### Refinement

*R*[*F* ^2^ > 2σ(*F* ^2^)] = 0.056
*wR*(*F* ^2^) = 0.149
*S* = 1.03
3060 reflections
192 parameters
14 restraints
H-atom parameters constrained
Δρ_max_ = 0.67 e Å^−3^
Δρ_min_ = −0.77 e Å^−3^

### 

Data collection: *SMART* (Bruker, 2007 ▶); cell refinement: *SAINT* (Bruker, 2007 ▶); data reduction: *SAINT*; program(s) used to solve structure: *SHELXTL* (Sheldrick, 2008 ▶); program(s) used to refine structure: *SHELXTL*; molecular graphics: *SHELXTL*; software used to prepare material for publication: *SHELXTL*.

## Supplementary Material

Crystal structure: contains datablocks global, I. DOI: 10.1107/S1600536808037902/ci2712sup1.cif

Structure factors: contains datablocks I. DOI: 10.1107/S1600536808037902/ci2712Isup2.hkl

Additional supplementary materials:  crystallographic information; 3D view; checkCIF report

## Figures and Tables

**Table 1:** Selected bond lengths (Å)

Ni1—N1	2.145 (4)
Ni1—N2	2.081 (4)
Ni1—N3	2.064 (5)
Ni1—N6	2.041 (4)
Ni1—N6^i^	2.175 (4)

Symmetry code: (i) 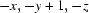.

**Fig. 1.**
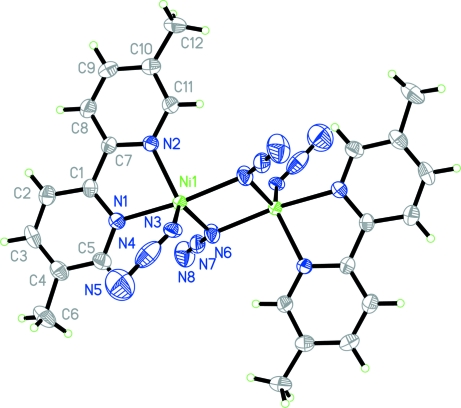
The molecular structure of the title compound, showing 30% probability displacement ellipsoids and the atom-numbering scheme.

## supplementary crystallographic information

### Comment

Polynuclear complexes play an important role in many fields, such as catalysis and magnetism (Dey *et al.*, 2007; Jiang *et al.*, 2005; Abramo *et al.*, 2002). The main strategy for the design of the polynuclear complexes is to use suitable bridging ligands. In this paper, we report the synthesis and molecular structure of the title azide-bridged dinuclear nickel(II) complex derived from 5,5'-dimethyl-[2,2']bipyridine.

The molecule of the title complex is located on a crystallographic centre of inversion (Fig. 1). The complex contains two NiL (L is 5,5'-dimethyl-[2,2']bipyridine) units connected to each other by two bridging azide ligands. The Ni^II^ atom in the complex is five-coordinated by two N atoms of 5,5'-dimethyl-[2,2']bipyridine ligand and by three N atoms from three azide ligands in a trigonal-bipyramidal geometry. The bond lengths subtended at the metal center are within normal ranges (Song *et al.*, 2007; Fu *et al.*, 2005). The Ni···Ni distance is 3.2398 (12) Å.

### Experimental

5,5'-Dimethyl-[2,2']bipyridine (2 mmol, 368.3 mg), sodium azide (4 mmol, 261.2 mg) and nickel acetate tetrahydrate (2 mmol, 497.8 mg) were dissolved in methanol (100 ml). The mixture was stirred for 30 min at room temperature to give a green solution. The solution was kept still in air for a week, green block-shaped crystals of the title complex were formed.

### Refinement

H atoms were positioned geometrically (C–H = 0.93-0.96 Å) and refined using a riding model, with *U*_iso_(H) = 1.2*U*_eq_(C) and 1.5*U*_eq_(C_methyl_). The displacement ellipsoids of atoms N4 and N5 are extremely elongated and hence the U^ij^ parameters of these atoms were restrained to an approximate isotropic behaviour. The distance between atoms N3 and N4 was restrained to 1.23 (1) Å and that between atoms N4 and N5 was restrained to 1.13 (1) Å.

### Crystal data

**Table:**

[Ni_2_(N_3_)_4_(C_12_H_12_N_2_)_2_]	*F*_000_ = 672
*M**_r_* = 654.01	*D*_x_ = 1.546 Mg m^−^^3^
Monoclinic, *P*2_1_/*n*	Mo *K*α radiation λ = 0.71073 Å
Hall symbol: -P 2yn	Cell parameters from 1576 reflections
*a* = 7.938 (2) Å	θ = 2.3–25.1º
*b* = 15.067 (3) Å	µ = 1.39 mm^−^^1^
*c* = 11.755 (2) Å	*T* = 298 (2) K
β = 91.650 (2)º	Block, green
*V* = 1405.3 (5) Å^3^	0.13 × 0.10 × 0.08 mm
*Z* = 2	

### Data collection

**Table:**

Bruker SMART CCD area-detector diffractometer	3060 independent reflections
Radiation source: fine-focus sealed tube	2165 reflections with *I* > 2σ(*I*)
Monochromator: graphite	*R*_int_ = 0.063
*T* = 298(2) K	θ_max_ = 27.0º
φ and ω scan	θ_min_ = 2.2º
Absorption correction: multi-scan(SADABS; Bruker, 2001)	*h* = −10→10
*T*_min_ = 0.840, *T*_max_ = 0.897	*k* = −19→19
11588 measured reflections	*l* = −15→15

### Refinement

**Table:**

Refinement on *F*^2^	Secondary atom site location: difference Fourier map
Least-squares matrix: full	Hydrogen site location: inferred from neighbouring sites
*R*[*F*^2^ > 2σ(*F*^2^)] = 0.056	H-atom parameters constrained
*wR*(*F*^2^) = 0.149	*w* = 1/[σ^2^(*F*_o_^2^) + (0.0643*P*)^2^ + 1.1062*P*] where *P* = (*F*_o_^2^ + 2*F*_c_^2^)/3
*S* = 1.03	(Δ/σ)_max_ = 0.001
3060 reflections	Δρ_max_ = 0.67 e Å^−^^3^
192 parameters	Δρ_min_ = −0.77 e Å^−^^3^
14 restraints	Extinction correction: none
Primary atom site location: structure-invariant direct methods	

### Special details

**Table:**

Geometry. All esds (except the esd in the dihedral angle between two l.s. planes) are estimated using the full covariance matrix. The cell esds are taken into account individually in the estimation of esds in distances, angles and torsion angles; correlations between esds in cell parameters are only used when they are defined by crystal symmetry. An approximate (isotropic) treatment of cell esds is used for estimating esds involving l.s. planes.
Refinement. Refinement of F^2^ against ALL reflections. The weighted R-factor wR and goodness of fit S are based on F^2^, conventional R-factors R are based on F, with F set to zero for negative F^2^. The threshold expression of F^2^ > 2sigma(F^2^) is used only for calculating R-factors(gt) etc. and is not relevant to the choice of reflections for refinement. R-factors based on F^2^ are statistically about twice as large as those based on F, and R- factors based on ALL data will be even larger.

### Fractional atomic coordinates and isotropic or equivalent isotropic displacement parameters (Å^2^)

**Table:**

	*x*	*y*	*z*	*U*_iso_*/*U*_eq_	
Ni1	0.03235 (7)	0.44079 (4)	0.11356 (5)	0.0386 (2)	
N1	0.2280 (5)	0.3549 (3)	0.1785 (4)	0.0491 (10)	
N2	−0.0777 (5)	0.3158 (3)	0.0994 (3)	0.0416 (9)	
N3	0.0029 (5)	0.5108 (3)	0.2626 (4)	0.0459 (11)	
N4	0.0958 (8)	0.5106 (4)	0.3252 (5)	0.0857 (18)	
N5	0.1983 (12)	0.5160 (6)	0.4040 (7)	0.144 (3)	
N6	0.1668 (5)	0.4913 (3)	−0.0170 (4)	0.0583 (12)	
N7	0.2901 (6)	0.4579 (3)	−0.0571 (4)	0.0594 (12)	
N8	0.4096 (8)	0.4282 (4)	−0.0950 (6)	0.094 (2)	
C1	0.1794 (6)	0.2706 (3)	0.1926 (4)	0.0445 (11)	
C2	0.2820 (7)	0.2113 (4)	0.2525 (5)	0.0573 (14)	
H2	0.2475	0.1529	0.2628	0.069*	
C3	0.4356 (7)	0.2398 (4)	0.2967 (5)	0.0632 (16)	
H3	0.5057	0.2000	0.3357	0.076*	
C4	0.4857 (6)	0.3259 (4)	0.2836 (5)	0.0575 (14)	
C5	0.3771 (6)	0.3811 (4)	0.2219 (5)	0.0570 (14)	
H5	0.4100	0.4396	0.2102	0.068*	
C6	0.6485 (7)	0.3623 (5)	0.3348 (5)	0.085 (2)	
H6A	0.6289	0.3847	0.4097	0.128*	
H6B	0.6886	0.4095	0.2878	0.128*	
H6C	0.7312	0.3159	0.3393	0.128*	
C7	0.0139 (6)	0.2474 (3)	0.1428 (4)	0.0442 (12)	
C8	−0.0506 (7)	0.1625 (4)	0.1383 (5)	0.0626 (15)	
H8	0.0127	0.1155	0.1680	0.075*	
C9	−0.2070 (8)	0.1468 (4)	0.0904 (5)	0.0652 (16)	
H9	−0.2501	0.0894	0.0893	0.078*	
C10	−0.3023 (6)	0.2160 (4)	0.0433 (5)	0.0530 (13)	
C11	−0.2308 (6)	0.2993 (3)	0.0520 (4)	0.0477 (12)	
H11	−0.2925	0.3472	0.0233	0.057*	
C12	−0.4711 (7)	0.2026 (4)	−0.0126 (5)	0.0657 (16)	
H12A	−0.5490	0.1824	0.0427	0.098*	
H12B	−0.4629	0.1591	−0.0719	0.098*	
H12C	−0.5103	0.2577	−0.0448	0.098*	

### Atomic displacement parameters (Å^2^)

**Table:**

	*U*^11^	*U*^22^	*U*^33^	*U*^12^	*U*^13^	*U*^23^
Ni1	0.0339 (3)	0.0328 (3)	0.0487 (4)	−0.0009 (3)	−0.0026 (2)	0.0057 (3)
N1	0.039 (2)	0.048 (3)	0.060 (3)	0.0018 (19)	−0.003 (2)	0.004 (2)
N2	0.039 (2)	0.040 (2)	0.046 (2)	0.0000 (17)	−0.0016 (18)	−0.0013 (17)
N3	0.035 (2)	0.036 (2)	0.067 (3)	0.0025 (18)	−0.006 (2)	−0.012 (2)
N4	0.119 (5)	0.043 (3)	0.097 (5)	0.001 (4)	0.045 (4)	−0.006 (3)
N5	0.165 (7)	0.149 (6)	0.115 (6)	−0.004 (6)	−0.029 (5)	0.002 (5)
N6	0.038 (2)	0.066 (3)	0.072 (3)	0.010 (2)	0.005 (2)	0.021 (2)
N7	0.051 (3)	0.056 (3)	0.070 (3)	0.005 (2)	−0.002 (2)	0.016 (2)
N8	0.071 (4)	0.105 (5)	0.108 (5)	0.037 (3)	0.020 (3)	0.011 (4)
C1	0.048 (3)	0.043 (3)	0.043 (3)	0.009 (2)	0.004 (2)	0.001 (2)
C2	0.065 (4)	0.048 (3)	0.058 (3)	0.010 (3)	0.002 (3)	0.002 (3)
C3	0.058 (4)	0.084 (4)	0.047 (3)	0.025 (3)	−0.009 (3)	0.004 (3)
C4	0.041 (3)	0.080 (4)	0.051 (3)	0.008 (3)	0.001 (2)	−0.001 (3)
C5	0.044 (3)	0.064 (4)	0.063 (4)	−0.003 (3)	−0.001 (3)	0.002 (3)
C6	0.049 (3)	0.131 (7)	0.074 (4)	−0.001 (4)	−0.014 (3)	0.003 (4)
C7	0.049 (3)	0.038 (3)	0.046 (3)	0.003 (2)	0.004 (2)	0.001 (2)
C8	0.060 (4)	0.047 (3)	0.080 (4)	0.003 (3)	−0.002 (3)	0.013 (3)
C9	0.067 (4)	0.047 (3)	0.081 (4)	−0.016 (3)	−0.001 (3)	0.004 (3)
C10	0.049 (3)	0.056 (3)	0.054 (3)	−0.008 (3)	0.006 (2)	−0.004 (3)
C11	0.045 (3)	0.047 (3)	0.050 (3)	0.001 (2)	0.001 (2)	0.002 (2)
C12	0.054 (3)	0.075 (4)	0.068 (4)	−0.020 (3)	−0.002 (3)	−0.009 (3)

### Geometric parameters (Å, °)

**Table:**

Ni1—N1	2.145 (4)	C3—H3	0.93
Ni1—N2	2.081 (4)	C4—C5	1.387 (7)
Ni1—N3	2.064 (5)	C4—C6	1.512 (8)
Ni1—N6	2.041 (4)	C5—H5	0.93
Ni1—N6^i^	2.175 (4)	C6—H6A	0.96
N1—C5	1.336 (6)	C6—H6B	0.96
N1—C1	1.339 (6)	C6—H6C	0.96
N2—C11	1.345 (6)	C7—C8	1.379 (7)
N2—C7	1.352 (6)	C8—C9	1.369 (8)
N3—N4	1.027 (6)	C8—H8	0.93
N4—N5	1.217 (7)	C9—C10	1.394 (8)
N6—N7	1.209 (6)	C9—H9	0.93
N6—Ni1^i^	2.175 (4)	C10—C11	1.380 (7)
N7—N8	1.149 (7)	C10—C12	1.489 (7)
C1—C2	1.387 (7)	C11—H11	0.93
C1—C7	1.465 (7)	C12—H12A	0.96
C2—C3	1.380 (8)	C12—H12B	0.96
C2—H2	0.93	C12—H12C	0.96
C3—C4	1.367 (8)		
			
N6—Ni1—N3	121.5 (2)	C3—C4—C6	123.2 (5)
N6—Ni1—N2	120.32 (18)	C5—C4—C6	120.1 (6)
N3—Ni1—N2	118.23 (17)	N1—C5—C4	123.5 (5)
N6—Ni1—N1	95.97 (17)	N1—C5—H5	118.2
N3—Ni1—N1	96.02 (17)	C4—C5—H5	118.2
N2—Ni1—N1	77.30 (15)	C4—C6—H6A	109.5
N6—Ni1—N6^i^	79.63 (18)	C4—C6—H6B	109.5
N3—Ni1—N6^i^	95.99 (18)	H6A—C6—H6B	109.5
N2—Ni1—N6^i^	94.98 (16)	C4—C6—H6C	109.5
N1—Ni1—N6^i^	167.78 (18)	H6A—C6—H6C	109.5
C5—N1—C1	119.3 (5)	H6B—C6—H6C	109.5
C5—N1—Ni1	125.6 (4)	N2—C7—C8	119.8 (5)
C1—N1—Ni1	114.1 (3)	N2—C7—C1	115.8 (4)
C11—N2—C7	119.0 (4)	C8—C7—C1	124.3 (5)
C11—N2—Ni1	124.9 (3)	C9—C8—C7	120.5 (5)
C7—N2—Ni1	116.1 (3)	C9—C8—H8	119.8
N4—N3—Ni1	120.7 (5)	C7—C8—H8	119.8
N3—N4—N5	174.4 (8)	C8—C9—C10	120.6 (5)
N7—N6—Ni1	125.8 (4)	C8—C9—H9	119.7
N7—N6—Ni1^i^	125.2 (4)	C10—C9—H9	119.7
Ni1—N6—Ni1^i^	100.37 (18)	C11—C10—C9	115.7 (5)
N8—N7—N6	178.1 (6)	C11—C10—C12	121.3 (5)
N1—C1—C2	120.5 (5)	C9—C10—C12	123.0 (5)
N1—C1—C7	115.8 (4)	N2—C11—C10	124.3 (5)
C2—C1—C7	123.8 (5)	N2—C11—H11	117.8
C3—C2—C1	119.3 (5)	C10—C11—H11	117.8
C3—C2—H2	120.3	C10—C12—H12A	109.5
C1—C2—H2	120.3	C10—C12—H12B	109.5
C4—C3—C2	120.7 (5)	H12A—C12—H12B	109.5
C4—C3—H3	119.7	C10—C12—H12C	109.5
C2—C3—H3	119.7	H12A—C12—H12C	109.5
C3—C4—C5	116.7 (5)	H12B—C12—H12C	109.5

Symmetry codes: (i) −*x*, −*y*+1, −*z*.
